# Research on combustion and emissions of heavy duty diesel engines under low-speed and low-load conditions in low temperature environments

**DOI:** 10.1371/journal.pone.0318933

**Published:** 2025-02-21

**Authors:** Jie Zhang, Gengfei Liu, Huaimin Xu

**Affiliations:** State Key Laboratory of Engines, Tianjin University, Tianjin, China; GH Raisoni College of Engineering and Management Pune, INDIA

## Abstract

China has a complex geographical environment, and in order to improve the combustion and emissions performance of heavy-duty diesel engines operating at low temperatures, low-speed and low-load conditions, a cleaner combustion mode has been proposed. This study utilizes a multi-cylinder engine environmental chamber test bench and CONVERGE 3D simulation software to examine the effects of intake temperature, injection timing, and injection pressure on engine combustion and emissions. The results indicate that increasing the intake temperature to 322 K, advancing the injection timing to 1 deg and raising the injection pressure to 120 MPa significantly improved the fuel-air mixture. These adjustments increased the OH radical content, accelerated oxidation cracking rates across low, medium, and high-temperature zones and effectively shortened combustion duration. Consequently, soot emissions were reduced by 23.1%, HC emissions by 33.3%, and CO emissions by 52.3%, demonstrating a significant improvement in combustion efficiency and a reduction in emissions from incomplete combustion.

## 1. Introduction

High thermal efficiency and low emissions have consistently been key goals in internal combustion engine research. Heavy-duty trucks, second only to light trucks, are significant contributors to global transportation emissions, and are primarily powered by internal combustion engines [1]. These engines produce harmful emissions, including hydrocarbons (HC), nitrogen oxides (NOx), carbon oxide (CO), particulate matter (PM), and lead compounds. If uncontrolled, these emissions pose significant risks to both human health and the environment [2]. From the perspective of China’s energy demand, petrochemical energy consumption continues to grow [3]. Moreover, China has implemented the National VI emission standards, equivalent to Euro VI, and is pushing forward stricter regulations. In order to meet the demands of future internal combustion engines, it is crucial to focus on improving combustion efficiency while reducing emissions [4]. Diesel engines operating under low-temperature and cold-start conditions face worsened combustion and emissions due to decreased fuel and intake temperatures, ultimately resulting in higher fuel consumption. Therefore, research on diesel engine combustion and emissions under low-temperature conditions remains a significant focus in the industry [5,6].

At present, the optimization of diesel engine combustion and emissions primarily focuses on three key aspects: intake temperature [7,8], injection pressure [9,10], and injection timing [11,12]. Numerous studies have investigated approaches to optimizing diesel engine combustion and emissions. During the initial operating cycles, the cylinder experiences low medium and fuel temperatures, resulting in a significant increase in the penetration distance of spray [13]. Additionally, the confined space within the cylinder often results in insufficient fuel atomization and spray impingement, contributing to increased particulate, NOx emissions and other problems [14,15]. Liu et al. [16] found that carbon soot emissions are significantly influenced by fuel injection pressure and nozzle aperture, which are primarily affected by the air intake during the injection process. Variations in air intake alter fuel-air mixing, thereby impacting carbon soot emissions. Consequently, the fuel-air mixing process is critical for emission generation. Mohamad [17] demonstrated that intake temperature is crucial for CO emissions. Increasing the intake temperature shortens the formation time of CO, while in this study, hydrogen addition raises the reaction temperature and reduces CO emissions. Yao [18] investigated the combustion process of diesel engines mixed with methanol under low-temperature conditions and found that raising intake temperature reduces HC and CO emissions. Wang et al. [19] used numerical simulations to assess the impact of intake thermodynamic conditions, showing that increasing intake pressure and temperature enhance atomization and evaporation, improving fuel mixing rates and thereby reducing heat loss and soot emissions in high equivalence ratio regions. This conclusion is further supported by Cinar et al. [20], who reported that increasing intake temperature from 40 °C to 120 °C raises cylinder gas temperature at the end of the compression stroke, diminishes the peak of low-temperature oxidation reaction, accelerate chemical reaction, increase cylinder pressure and heat release rate, advance combustion timing, and shorten the combustion duration. Capacitance tests performed by Ma [21] on varying background temperatures revealed that lower background temperatures displace the ignition point further from the nozzle and significantly weaken the flame. These findings collectively indicate that higher temperatures improve diesel combustion. In addition, SIEBERS et al. [22] found through the study of fuel elasticity that in addition to increasing temperature, increasing the injection pressure can also improve the combustion characteristics of diesel, and the peak value of soot will decrease with the increase of injection pressure. With the increase of fuel injection pressure, the reduction in soot is proportional to the square root of the pressure drop at the injector nozzle, mainly due to increased air intake by the fuel jet. Using LIEF-PIV, Wang et al. [23] conducted experimental analysis on the concentration field and velocity field of low-temperature fuel at 20 ~ 40 °C with an injection pressure range of 40 ~ 80 MPa. The results indicated that higher injection pressure increased the initial kinetic energy of diesel droplets, reduced the concentration gradient of the spray, and enhanced mixing with ambient gases. Similarly, Dave et al. [24] found that increasing the injection pressure from 500 bar to 700 bar resulted in a more uniform fuel-air mixture, improved overall combustion conditions, and significantly reduced HC, soot emissions, and fuel consumption. Wang et al. [25] investigated the effects of a microporous nozzle (diameter 0.08mm) and ultra-high injection pressure (300 MPa) on diesel flame structure and soot emissions using a constant-volume combustion bomb. Their research results showed that no soot was detected when injection pressure exceeded 200 MPa with a microporous nozzle. A micro hole nozzle will shorten the penetration distance of spray and reduce the possibility of spray impingement on the wall, which can also greatly reduce soot emissions. Besides intake temperature and injection pressure, injection timing has also been extensively studied [26–28]. You et al. [29] observed that advancing injection timing enhances the chemical kinetics of diesel ignition, resulting in increased concentration and distribution of the highly active component CH_2_O, as well as a gradual rise in low-temperature zones. A strong correlation exists between high chemical activity and low-temperature combustion. Lu [30] studied the impact of the precombustion chamber jet on the combustion in the main combustion chamber. The study found that during the later stages of combustion, the kinetic energy of the jet transfers into the main chamber, causing oil film detachment from the walls. This process facilitates better mixing, which enhances ITEg and further reduces smoke and CO emissions. Duan et al. [31] also observed that delaying the SOI deteriorates combustion efficiency and emission profiles. Similarly, the study by F Okumuş [32] showed that delayed injection timing leads to increased HC and CO emissions.

Previous research indicates that the fuel-air mixture significantly affects engine combustion and emissions. However, studies on low emissions from diesel engines operating in low-temperature environments have primarily focused on single parameters, lacking a comprehensive analysis of the impact of multi-parameter coupling on engine combustion and emissions, and the development of clean combustion strategies. The goals are to reduce reliance on foreign engine technology, improve engine performance, and explore cleaner combustion modes. This study controlled intake air temperature, diesel injection timing, and injection pressure to systematically analyze their the effects on fuel-air mixing, surface spray atomization, chemical reaction kinetics, and pollutant formation. This study provides mathematical support and empirical reference for advancing clean combustion theory.

## 2. Materials and methods

### 2.1. Computational models

**Fig 1:**
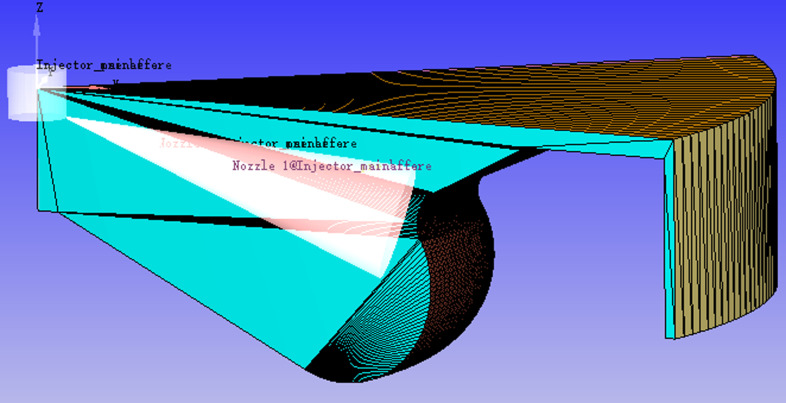
The configuration of the computational model.

Fig 1 presents the configuration of the computational model. Due to the rotational symmetry of the combustion chamber and the uniform circumferential distribution of the 8 injection holes, one-eighth of the chamber is modeled to improve computational efficiency. The engine speed is 1000 r/min, and the fuel injection amount is 85 mg/cyc. The pre injection timing is set at −18.5 deg, while the main injection timing is 3 deg. The angle between the nozzle and the centerline is 73 degrees. The intake valve closes at −146 deg, and the exhaust valve opens at 121 deg. The parameters of the test engine are provided in Table 1.

**Table 1:** Main technical parameters of heavy-duty diesel engine.

Parameter	Value
Cylinder diameter/ mm	127
Stroke/ mm	165
Displacement/ L	12.8
Compression ratio	18
Intake mode	Turbocharged and Intercooled
Protrusion height/ mm	2.53
Nozzle number × Diameter	8 × 0.217

### 2.2. Computational numerical simulation methods and boundary models

Before the simulation calculation under low temperature conditions, the calculation model of 1000 r/min and 30% load condition was calibrated to ensure the accuracy of the calculation model. Prior to the calibration, the following assumptions were made: the entire engine was assumed to operate in a specific low temperature environment, and the engine cooling system, the intake system and the internal temperature of the engine were assumed to be kept within a certain range at the ambient temperature. According to the test data, the 3D computational simulation model was calibrated and verified, and the boundary conditions used were shown in Table 2.

**Table 2.** Model calibration boundary parameter.

Boundary parameter	Value
Rotational speed	1000 r/min
Torque	690 N·m
Injection timing	−18.5 °CA ATDC; Pre-spray
	3 °CA ATDC; Main spray
Injector out height	2.53 mm
Orifice diameter	0.000217 m
Angle between the nozzle and the center line of the cylinder	73 °
Number of holes	8
Fuel injection quantity	85 mg
Intake valve closing time (IVCT)	−146 °CA ATDC
Exhaust valve opening time (EVOT)	121 °CA ATDC
Temperature of piston	520K
Temperature of wall	470K
Temperature of cylinder head	500K

The various physical and chemical models incorporated into the computational model are summarized in Table 3. The governing equations for all models are solved by CONVERGE. The characterization fuel of gasoline is heptane (C7H16), and the chemical reaction mechanism employed is a 42-component, 142-step simplified model. The simulation uses a basic grid size of 4 mm, enhanced by an adaptive mesh refinement technique, with a minimum grid size of 0.5 mm, and a maximum grid count of approximately 1 million.

**Table 3:** Physical and chemical models of simulation.

Process	Model
Turbulence	RNG k-ε
Spray break	KH-RT
Spray-wall interaction	Rebound/slide
Fuel collision	NTC
Drop evaporation	Frossling
NOx emission model	Extended Zeldovich NOx
soot Emission Model	Hiroyasu
Wall heat transfer	Han and Reitz
Combustion	SAGE chemical reaction solver & Simplify chemical reaction mechanism

The chemical reactions based on the complete mechanism can be solved for CO and HC pollutants. The following text provides a brief introduction to the emission models for NOx and soot.

CONVERGE can output the materials of each computational grid as needed, which provides great assistance in analyzing the impact of various combustion control measures on emission generation and oxidation. A reasonable emission model is crucial for accurately describing and predicting engine emission performance. In this study, the extended Zel’dovich NOx emission model [33] was selected, which mainly includes three chemical reactions:


$$O+N_{2}\Leftrightarrow NO+N$$ (k1)

$$N+O_{2}\Leftrightarrow NO+O$$ (k2)

N+OH⇔NO+H (k3)

In the equation, k1, k2, and k3 are chemical reaction constants.

The Hiroyasu soot generation model selected in this study is a modified two-step model, which mainly includes gas-phase reaction kinetics and solid particle kinetics. The final soot emissions are the result of the combined effect of carbon particle growth and oxidation consumption.

The quality growth rate of soot is:


$${\overset{\cdot }{M}}_{sf}=\mathrm{SF}M_{\mathrm{form}}$$

$$SF=A_{sf}P^{0.5}e^{\left(\frac{-E_{sf}}{R_{u}T}\right)}$$

In the formula, ${\overset{\cdot }{M}}_{sf}$ represents the mass growth rate of soot, *M*_*form*_ represents the mass of soot products, *A*_*sf*_ is the pre exponential factor, *E*_*sf*_ is the activation energy of the reaction, and *R*_*u*_ is the gas constant.

The oxidation rate of soot is:


$${\overset{\cdot }{M}}_{so}=SR_{\mathrm{total}}{\mathrm{MW}}_{c}$$

$$S=N_{p,soot}\pi D_{s}^{2}=\frac{6M_{s}}{\rho_{s}D_{s}}$$

$$R_{total}=\left(\frac{K_{A}P_{O_{2}}}{1+K_{Z}P_{O_{2}}}\right)X+K_{B}P_{O_{2}}\left(1-X\right)$$

$$X=\frac{P_{O_{2}}}{P_{O_{2}}+\left(\frac{K_{T}}{K_{B}}\right)}$$

In the formula, ${\overset{\cdot }{M}}_{so}$ represents the oxidation rate of soot, *R*_*total*_ is the net reaction rate, *MW*_*C*_ is the atomic mass of carbon, *N*_*p, soot*_ is the total number of soot particles, *Ds* is the average diameter of soot particles, *Ms* is the total mass of soot, ρsrepresents soot density, *K*_*A, B, T, Z*_ are reaction constants, and $P_{O_{2}}$is the partial pressure of oxygen in the atmosphere.

### 2.3. Experimental validation

**Fig 2:**
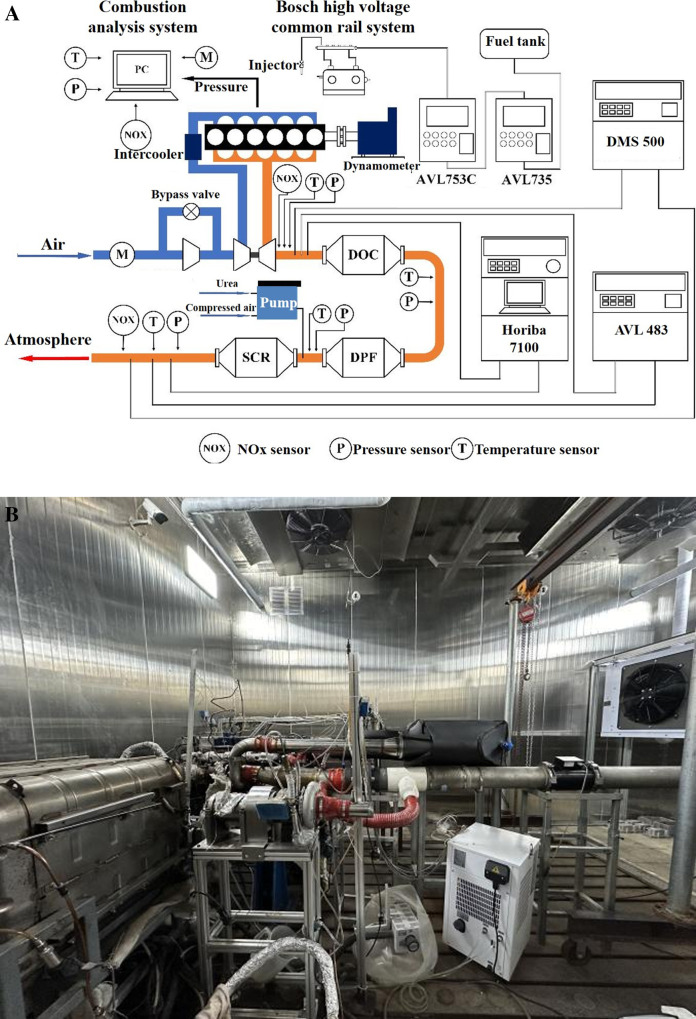
Schematic diagram and physical image of the experimental system: (a) schematic diagram; (b) physical image.

Fig 2 provides both the schematic diagram and a physical image of the experimental system. The system consists of a turbocharged intake system, an electronic control system, a high pressure common rail fuel supply system, a data acquisition and monitoring system, a multifunctional combustion parameter acquisition and analysis system, and a composition of exhaust emission testing system, etc.

The control system, developed independently by our research group, enables precise and consistent regulation of the intake, fuel injection, and ignition processes. The data acquisition system, a self-editing module based on the LabVIEW platform, allows for flexible adjustment of acquisition frequency and quantity as needed. This ensures that cylinder pressure is collected accurately.

To meet the analysis of emissions performance, a corresponding emission data collection system was built. The HORIBA 7100 gas analyzer is used to analyze the composition of exhaust gas. The analyzer includes a non dispersive infrared analyzer (NDIR) for measuring CO_2_ and CO, a chemiluminescence analyzer (CLD) for measuring NOx, and a heated hydrogen flame ionization chemical analyzer (FID) for measuring HC. AVL483 smoke meter is used to measure carbon smoke emissions, and DMS500 rapid particle spectrometer is used to analyze the particle size distribution of emitted particles.

**Fig 3:**
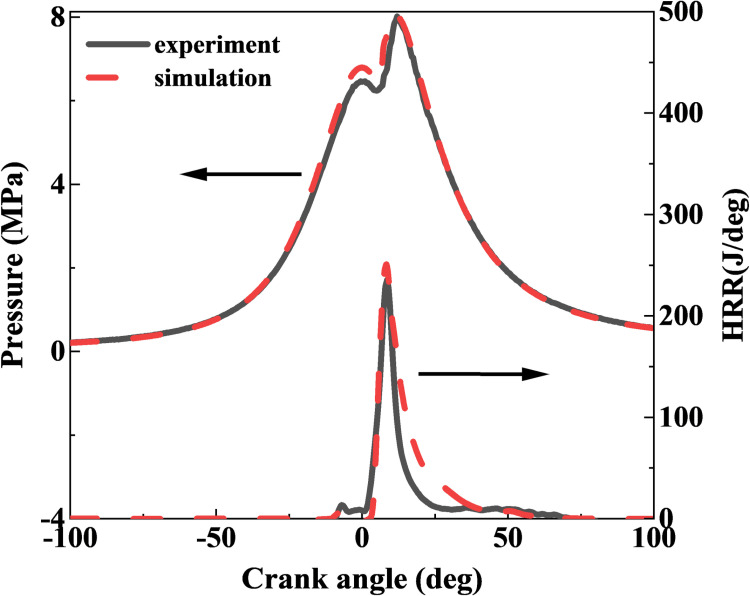
Comparison of pressure and HRR between experimental results and numerical simulation.

Fig 3 presents the comparison curve of cylinder pressure and heat release rate (HRR). Grid independence was verified by reducing the grid size by half, with the error found to be under 3%, indicating that the basic grid size of 4 mm meets computational requirements. The shape of the cylinder pressure and heat release rate curves derived from simulations and experiments showed strong consistency. The error in the peak cylinder pressure between simulation and experiment is less than 0.5%, and the error in the crankshaft angle corresponding to this peak does not exceed 0.1 deg. The discrepancy in the peak value of the heat release rate curve is under 5%. However, certain discrepancies exist between the simulation and experimental data during the pressure drop phase, attributable to the inherent return hysteresis of the piezoelectric sensor. In summary, the numerical simulations conducted in this study effectively capture the ignition and detonation processes of PDC, demonstrating their suitability for investigating various parameters.

Table 4 displays the comparison results of different emission data, which were obtained through an error analysis conducted using emission data from both experiments and simulations, aimed at further assessing the accuracy of the simulated data.

**Table 4.** Comparison of emission datas between simulation and experiment.

	Test	Simulation	Error
NO_x_ (g/kWh)	2.412	2.364	−2%
Soot (g/kWh)	0.014	0.013	−2.08%
HC (g/kWh)	0.044	0.045	2.3%
CO (g/kWh)	0.087	0.085	−2.3%

## 3. Results

### 3.1. The influence mechanism of intake temperature on combustion and emissions

In this study, the ambient temperature is maintained at 258 K, with an injection pressure of 100 MPa and an engine speed of 1000 r/min. The intake temperatures are set at 282, 302, 322, and 342 K, representing typical post-intercooling intake temperatures in diesel engines. Intake temperatures that are excessively high can lead to reduced air charge.

**Fig 4:**
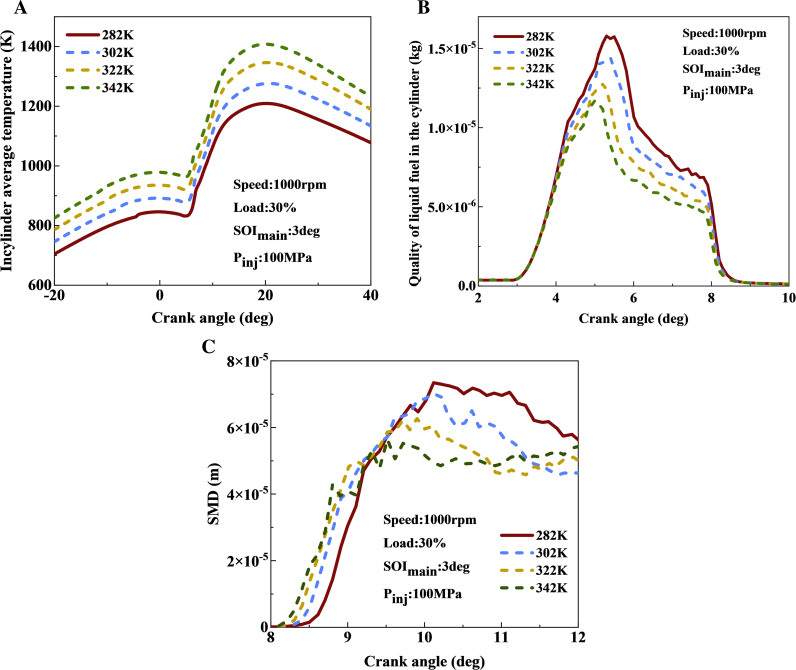
Influence of intake air temperature on average in-cylinder temperature and spray characteristics: (a) average cylinder temperature; (b) quality of liquid fuel; (c) SMD.

Fig 4 presents the influence of intake air temperature on the average temperature in cylinder temperature and spray development at 1000 r/min and 30% load. Figs 4a and 4b demonstrate that as the intake temperature increases, the average temperature rises. For every 20 K increase in intake temperature, the peak average temperature inside the cylinder increases by approximately 50 K. This rise in intake temperature elevates the initial background temperature within the cylinder, promoting rapid fuel evaporation. Consequently, the mass of liquid fuel within the cylinder decreases at various degrees with increased intake temperature, elevating the combustion chamber’s background temperature, which favors the evaporation process.Increasing the intake temperature from 282 K to 342 K reduces the mass of liquid fuel in the cylinder by approximately 20%. This finding aligns with the previous analysis that an elevated intake temperature is beneficial for accelerating fuel evaporation. Therefore, as the intake temperature increases, the mass of liquid fuel in the cylinder decreases and the proportion of fuel evaporation increases. The fuel evaporation process significantly impacts the subsequent combustion behavior.

Fig 4c illustrates the effect of different intake temperatures on droplet sauter mean diameter (SMD) within the cylinder at 1000 r/min and 30% load. As the intake temperature increases, the average temperature inside the cylinder continues to rise, enhancing the fuel evaporation rate and consequently reducing the average droplet diameter.As the intake temperature increased from 282 K to 342 K, the SMD peak decreased by approximately 28.6%.

**Fig 5:**
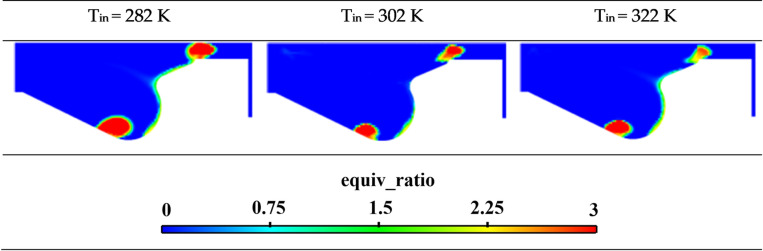
Cloud map of equivalent ratio distribution within the cylinder at different intake temperatures.

Fig 5 illustrates the equivalence ratio within the cylinder at different intake temperatures at 12 deg moments. It can be observed that as the intake temperature increases, the extent of the rich zone within the cylinder decreases significantly. This effect is attributed to the enhanced fuel evaporation rate and more thorough fuel-air mixing at higher intake temperatures, resulting in the formation of more lean mixtures.

**Fig 6:**
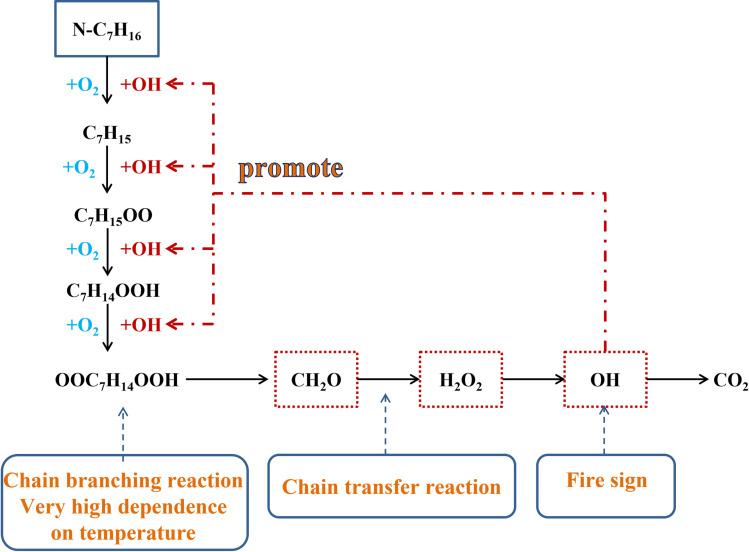
Schematic diagram of the diesel oxidation mechanism.

Fig 6 presents a schematic diagram of the diesel oxidation mechanism, illustrating the oxidation process of n-heptane as a diesel substitute. The oxidation of n-heptane strongly depends on temperature, with distinct reaction pathways and products across varying temperature ranges. The temperature range is divided into three zones: low, medium, and high temperature. The transition temperature between these zones varies based on the type of hydrocarbon fuel and environmental pressure. Based on the following diagram, each zone is described in detail.

In the low-temperature zone, the primary reactions are the dehydrogenation of n-heptane molecules and its initial oxygenation. Initially, n-heptane undergoes dehydrogenation and oxygenation with molecular oxygen. When other free radicals such as OH, O, and H appear in the reaction system, they significantly enhance dehydrogenation, with OH radical being particularly effective. Dehydrogenation is followed by the reaction of alkyl groups with oxygen to form peroxyalkyl radicals, the key reaction in the low-temperature oxidation stage, whose rate is highly temperature-dependent. Further along the low-temperature pathway, isomerization converts peroxyalkyl radicals to its isomers, followed by secondary oxygenation. The resulting OOC_7_H_14_ dissociates into stable intermediates, such as hydrogen peroxide ketones and OH. When the temperature exceeds 800 K, hydrogen peroxide ketone dissociates, producing a large number of radicals. As long as the temperature is high enough to cause the dissociation of hydrogen peroxide ketone, the generation of a large number of free radicals will lead to rapid chain branching reactions.

The low-temperature reaction is exothermic, raising the system temperature by several hundred degrees. Thus, this oxidation stage is also referred to as “cool flame” combustion. The two main roles of this stage are to increase system temperature through heat release and to generate initiating radical groups, which facilitate chain branching and subsequent reactions at higher temperatures. Here, CH_2_O is chosen as a representative product of low-temperature reactions.

When the system temperature rises to a certain level, reactions in the medium-temperature zone become more important than those in the low-temperature zone. The chain branching reaction in the low-temperature region are gradually replaced by chain transfer reaction in the medium-temperature region. The reaction in the medium-temperature zone mainly occurs as chain transfers, reducing the system reactivity and leading to the NTC phenomenon. Additionally, the primary significance of the medium-temperature zone is the accumulation of H_2_O_2_:


$$RH+HO_{2}\Rightarrow R+H_{2}O_{2}$$

With the sufficient energy released in the medium-temperature zone, the system temperature rises, so the system moves from the reactions in the medium temperature zone to the reaction in the high temperature zone. When the temperature exceeds 1200 K:


$$\cdot H+O_{2}\to \cdot O+\cdot OH$$

This reaction is a chain branching reaction with a decisive role in combustion. Another key reaction in the high-temperature zone is the dissociation of H_2_O_2_ into two hydroxyl radicals. The chain branching reaction occurs at a temperature of approximately 1000 K, and its significance lies in the fact that it is a sign of ignition:


$$H_{2}O_{2}+M\to \cdot OH+\cdot OH+M$$

The generated OH rapidly reacts with the fuel, causing a sharp increase in system temperature which is the occurrence of ignition. Additionally, the OH component significantly promotes the oxidative cracking of fuel and further oxidation of soot.

Based on the analysis of the combustion mechanism of n-heptane, the occurrence of reactions in the low-temperature, medium temperature, and high-temperature regions has a crucial impact on the combustion of n-heptane. This study identifies CH_2_O as the signature component for low-temperature reactions, H_2_O_2_ for medium-temperature reactions, and OH for high-temperature reactions. The analysis of the combustion process is based on these characteristic components.

**Fig 7:**
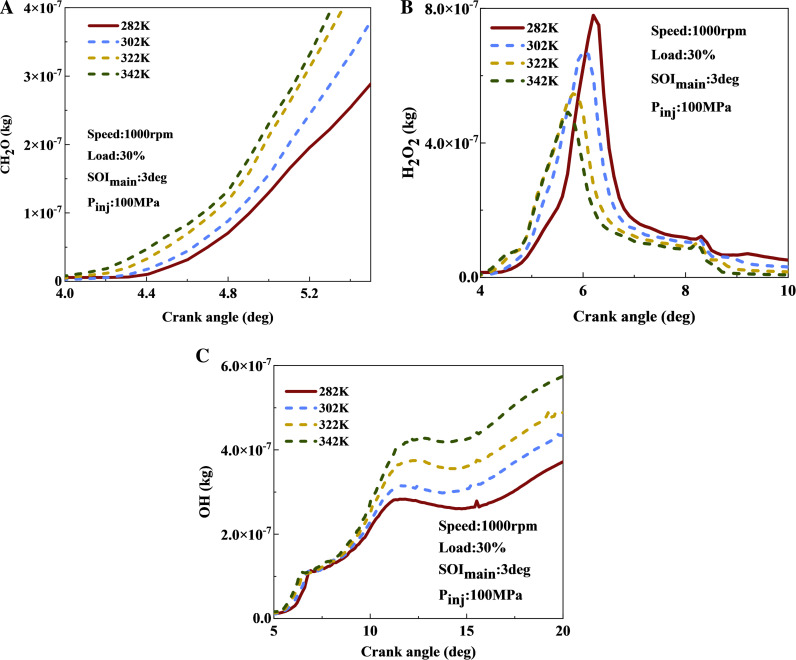
The effect of intake temperature on CH_2_O, H_2_O_2_, and OH contents: (a) CH2O; (b) H2O2; (c) OH.

Fig 7 illustrates the effect of intake temperature on the concentrations of CH2O, H2O2, and OH. Elevating the intake temperature within a certain range promotes the accumulation of more CH2O components, thereby facilitating rapid low-temperature reactions. In Fig 7b, an increase in intake temperature leads to a rapid generation of H2O2. The peak concentration of H2O2 varies across different intake temperatures: with an increase in intake temperature, the peak value decreases and its occurrence shifts earlier. As the intake temperature increased from 282 K to 342 K, the peak H2O2 concentration decreased by approximately 30%, with the peak occurring 0.5 degrees earlier. This is because the formation of H2O2 requires specific temperature conditions, so increasing the intake temperature accelerates its accumulation. As the temperature inside the cylinder further increases, H2O2 decomposes, which requires a temperature above 1000 K. Under higher intake temperatures, the cylinder reaches the decomposition temperature of H2O2 earlier, resulting in a quicker high-temperature reaction, specifically the decomposition of H₂O₂ into OH. Therefore, a higher intake temperature advances the peak of H2O2, indicating that the fuel is more likely to transition from the medium-temperature to the high-temperature zone under these conditions. According to Fig 7c, the OH content significantly increases with rising intake temperature. At 20 deg, the OH content is 60% higher at an intake temperature of 342 K compared to 282 K. The abundant presence of OH is indicative of a smooth transition of fuel reactions into to the high-temperature zone. Therefore, as the intake temperature increases, the reaction intensifies across all zones: low-temperature zone, medium temperature zone, and high-temperature zone.

**Fig 8:**
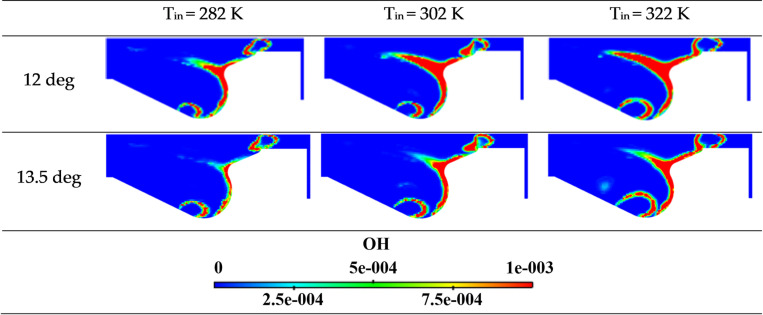
Cloud map of OH mass fraction at different intake temperatures.

Fig 8 provides a comparison of OH component cloud maps at 12 and 13.5 deg time. The distribution maps illustrate that, with increasing intake temperature, both the range and high-concentration areas of OH components within the cylinder expand significantly.

**Fig 9:**
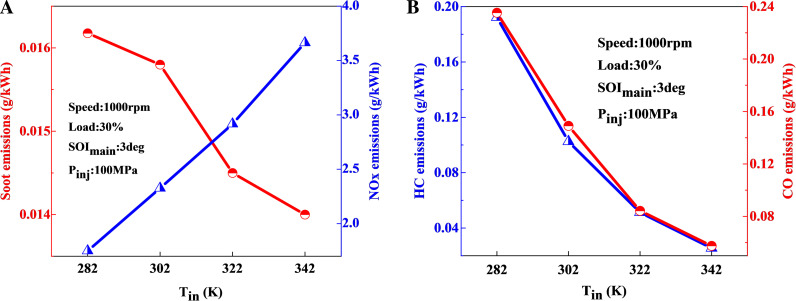
Impact of intake temperature on emissions: (a) soot-NOx; (b) HC-CO.

Fig 9 illustrates the impact of intake temperature on emissions. As the ambient temperature increases, NOx emissions exhibit a monotonic increase trend, attributed to the corresponding increase in the average in-cylinder temperature. In contrast, emissions of incomplete combustion products, such as soot, HC, and CO, display an overall decreasing trend with increasing intake temperature. This reduction is due to a higher proportion of lean mixtures and elevated OH concentrations at higher temperatures. Since OH is a crucial free radical involved in the subsequent oxidation of soot and other incomplete combustion products, its increased presence leads to reduced emissions of these products as the intake temperature increases.

Considering the energy consumption required to increase the intake temperature and its effect on emissions reduction, the results indicate that under conditions of 1000 r/min and 30% load conditions, increasing the intake temperature to 322 K from an ambient temperature of 258 K leads to a reduction of soot by 10.5%, 68.4% in HC emissions, 61.2% in CO emissions, and an increase of 111.4% in NOx emissions.

### 3.2. The influence mechanism of main injection timing on combustion and emissions

Based on of an intake temperature of 322 K, the original main injection timing (SOI_main_) was set at 3 deg. To evaluate the effects of varying injection timings, the timing is adjusted to −3, −1, 1, 5, and 7 deg.

**Fig 10:**
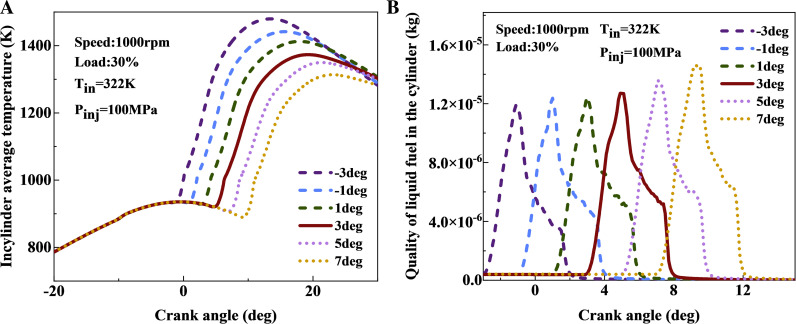
Comparison curve of average cylinder temperature and liquid fuel mass within the cylinder under different fuel injection timings: (a) average cylinder temperature; (b) quality of liquid fuel.

According to Fig 10a, as the fuel injection timing is delayed, the average temperature inside the cylinder decreases significantly, potentially deteriorating the thermal environment inside the cylinder, A 1 deg delay in the main injection timing reduces the peak average cylinder temperature by approximately 30 K. However, advancing the fuel injection timing markedly improves both the temperature inside the cylinder and the duration of high temperature. Fig 10b shows the comparison curve of liquid fuel mass within the cylinder at different injection timings. Advancing the fuel injection timing causes an earlier rise in cylinder temperature, with the peak value of liquid fuel mass showing a decreasing trend. The peak mass of liquid fuel decreases by about 2% for each 1 deg advance in injection timing. However, when the timing is advanced beyond 3 deg, the rate of decrease becomes less pronounced. The rise in average cylinder temperature favors the evaporation process of liquid fuel, thus reducing the peak mass of accumulated liquid fuel as the injection timing advances.

**Fig 11:**
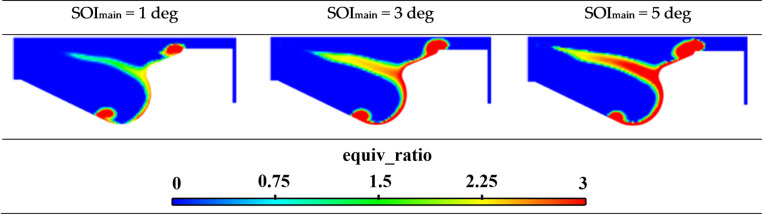
Cloud chart of equivalence ratio for different fuel injection timings.

Fig 11 presents the equivalence ratio distribution cloud map at different injection timings. To avoid redundancy, the cloud maps display the main injection timing at 1, 3, and 5 deg, followed by an injection timing of 7.5 deg. According to the comparison of these cloud maps reveals that, after fuel injection is initiated and at the same crankshaft angle, the rich zones within the cylinder increase significantly. Late fuel injection timing leads to lower cylinder temperatures and a gradual decrease in compression ratio during fuel injection, which hinders efficient combustion. In contrast, early fuel injection timing promotes the formation of a leaner mixture in the cylinder, benefiting the the subsequent combustion process.

**Fig 12:**
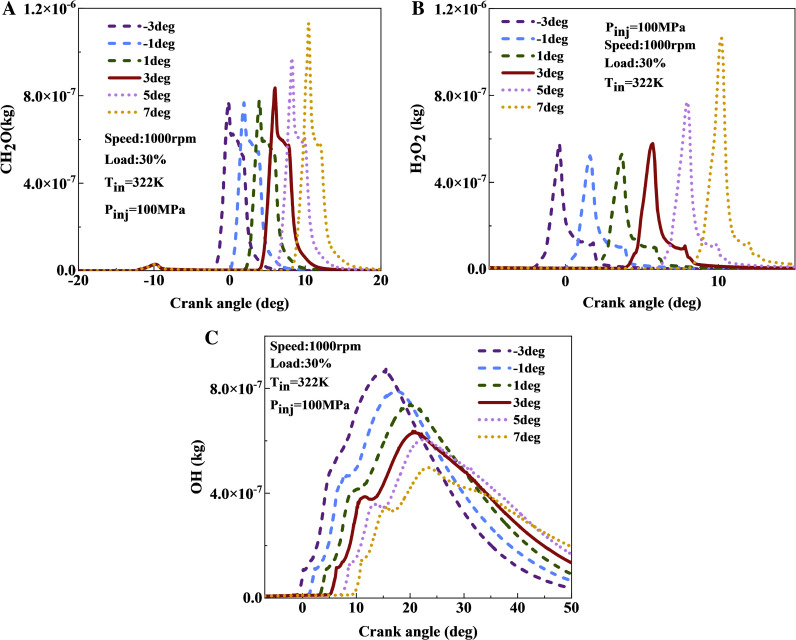
The effect of fuel injection timing on CH_2_O, H_2_O_2_, and OH coments: (a) CH_2_O; (b) H_2_O_2_; (c) OH.

Fig 12 shows a comparison of variations in the content of CH_2_O, H_2_O_2_, and OH radicals content in the cylinder across different main injection timings. Fig 12a indicates that as the main injection timing advances, the generation of CH_2_O occurs earlier, while the peak CH_2_O concentration increases with delayed main injection timing. Advancing the main injection timing from 7 to 3 deg decreases the CH_2_O peak by 18%, while between −3 and 1 deg, the peak value of CH_2_O remains almost unchanged. This delay results in lower cylinder temperatures during fuel injection, hindering fuel evaporation, reducing lean mixture content, and negatively impacting subsequent oxidation and decomposition reactions. As shown in Fig 12b, the peak concentration of H_2_O_2_ increases with delayed fuel injection timing. This is because the transition of fuel from the medium-temperature to the high-temperature zone is impeded, causing substantial accumulation of H_2_O_2_ within the cylinder. Fig 12c demonstrates that the peak OH concentration significantly increases as the main injection timing advances. When the injection timing is advanced from 7 to −3 deg, the peak OH content increases by approximately 55%. The advancement of injection timing enables a smooth progression of fuel through low-temperature and medium-temperature reactions, subsequently transitioning to high-temperature reactions, which results in the production of a large quantity of OH radicals.

**Fig 13:**
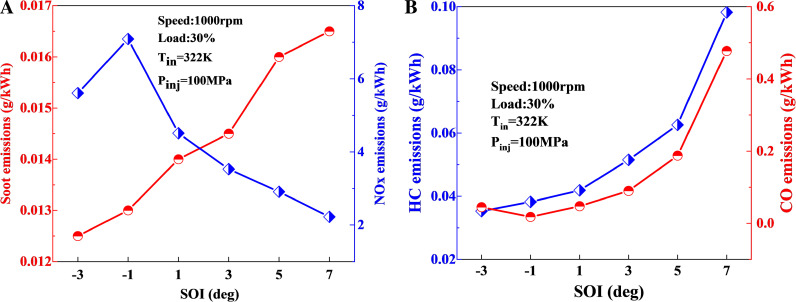
The impact of SOI_main_ on emissions: (a) soot-NOx; (b) HC-CO.

The impact of main injection timing on emissions is shown in Fig 13. The comparison reveals that as the main injection timing advances, NOx emissions display an overall increasing trend, while soot, HC, and CO emissions exhibit an overall decreasing trend. The increase in NOx emissions is associated with the elevated OH content within the cylinder, which serves as an indicator of high-temperature reactions. A higher concentration of OH corresponds to elevated temperatures and a broader high-temperature zone, leading to increased NOx formation. Additionally, the presence of a substantial amount of OH promotes more complete combustion and oxidation of the fuel, resulting in reduced soot, HC, and CO emissions as the injection timing advances. Before advancing the main injection timing to 1 deg, the rate of reduction in HC and CO emissions slows down, whereas the increase in NOx emissions accelerates. Therefore, the optimal main injection timing for this operating condition is chosen as 1 deg.

Based on the optimization results for fuel injection timing, advancing the main injection timing to 1 deg, as compared to 3 deg, results in a reduction of soot by 3.4%, HC emissions by 28.3%, and CO emissions by 47.1%, while NOx emissions increase by 13.5%.

### 3.3. The influence mechanism of injection pressure on combustion and emissions

Building on an intake temperature of 322 K and a main injection timing of 1 deg, the injection pressure is increased to 110, 120, 130, and 140 MPa. Since 140 MPa is the maximum achievable injection pressure under low-load conditions for this machine, it is selected as the upper limit.

**Fig 14:**
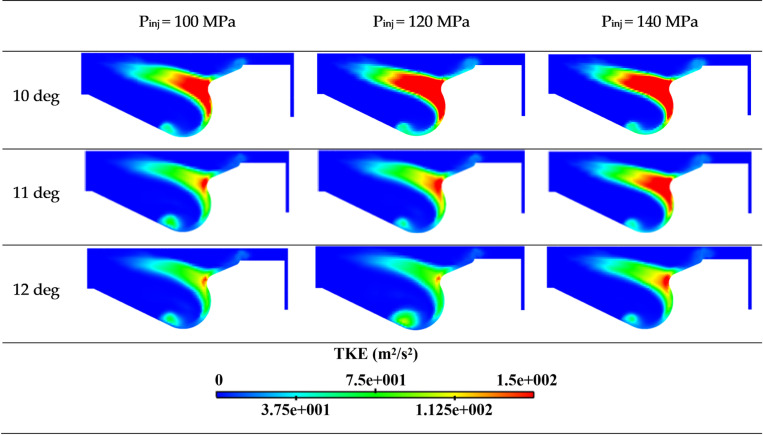
Cloud map of turbulent kinetic energy distribution under different fuel injection pressures.

Fig 14 presents the cloud map of turbulent kinetic energy within the cylinder at different injection pressures at 10, 11 and 12 deg. Turbulent kinetic energy serves as an important indicator of the turbulent mixing ability within the cylinder. As the fuel injection pressure increases, the proportion of regions with high turbulence kinetic energy areas also increases. High turbulence energy enhances fuel and gas mixing, thereby accelerating the rate of diesel and gas mixing.

**Fig 15:**
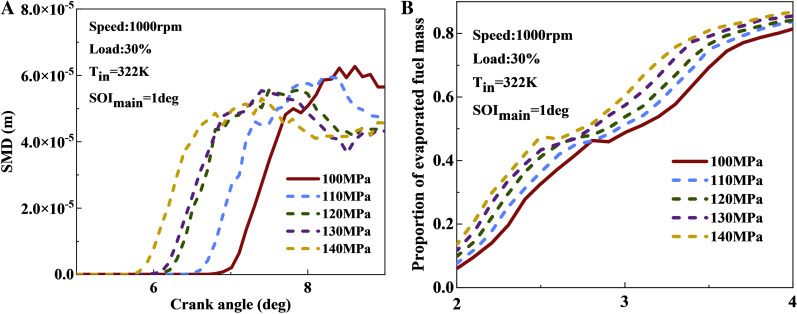
Spray characteristics under different injection pressures: (a) SMD; (b) proportion of evaporated fuel mass.

Fig 15 shows the comparison curve of changes in SMD and the proportion of fuel evaporation mass. From Fig 15a, it can be seen that as fuel injection pressure increases, the fuel beam preferentially breaks, resulting in a smaller SMD. For every 10 MPa increase in injection pressure, the fuel evaporation rate rises by 10% to 20%. This is due to the increased momentum of the fuel spray at higher injection pressures, leading to more intense collisions between the fuel spray and surrounding air molecules, which facilitates the breakup into finer droplets. Therefore, SMD gradually decreases with higher fuel injection pressure. Fig 15b illustrates that increasing the fuel injection pressure enhances fuel evaporation. This is because the reduction in droplet SMD increases the surface area in contact area with air, which is beneficial for accelerating its evaporation rate.

**Fig 16:**
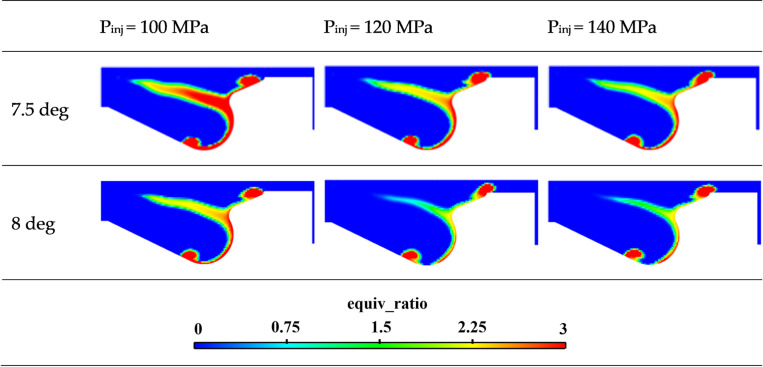
Cloud map of distribution for equivalence ratios at different fuel injection pressures.

Fig 16 illustrates the equivalence ratio at 7.5 and 8 deg time under different injection pressures. According to the distribution of the equivalence ratio cloud map, it is evident that the rich zone within the cylinder decreases significantly as injection pressure increases. The increase in fuel injection pressure enhances turbulence energy, decreases fuel SMD, increases the proportion of evaporated fuel, and promotes more thorough fuel-air mixing, resulting in a higher proportion of lean mixtures.

As fuel injection pressure increases, both the evaporation of fuel in the cylinder and the diameter of oil droplets improve, resulting in the formation of a more lean mixture in the cylinder.

**Fig 17:**
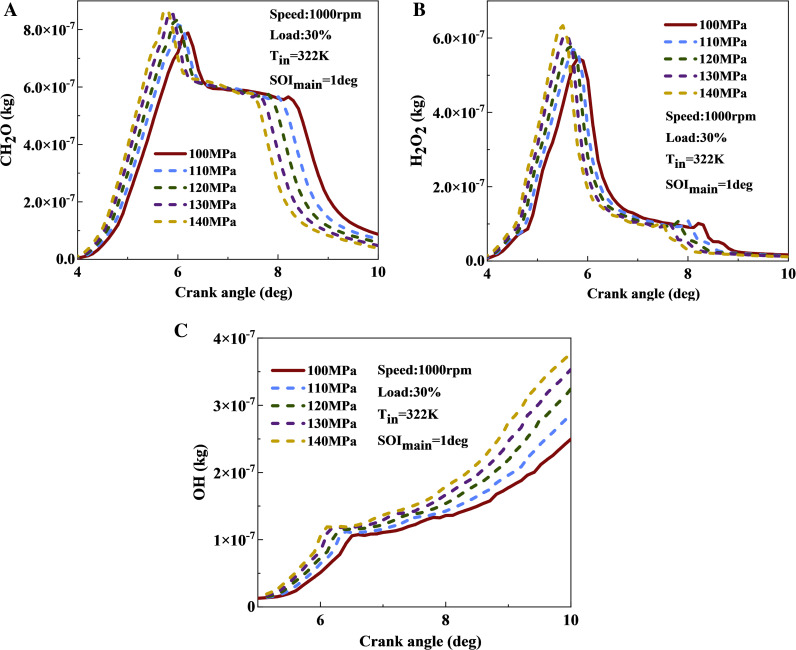
The effect of fuel injection pressure on CH_2_O, H_2_O_2_, and OH contents: (a) CH_2_O; (b) H_2_O_2_; (c) OH.

Fig 17 illustrates the effect of fuel injection pressure on combustion intermediate components. With increasing injection pressure, the accumulation of CH_2_O components gradually increases. Increasing the injection pressure from 100 MPa to 140 MPa increases the peak value of CH_2_O increased by approximately 10.4%. This occurs because the higher injection pressure breaks the fuel spray into finer droplets, accelerates the evaporation rate, increases the mass of gaseous fuel, and enhances the proportion of lean mixtures. Therefore, more fuel can participate in low-temperature reactions resulting in greater CH_2_O accumulation. The peak value of CH_2_O component also increases with the increase of injection pressure. This indicates that under high injection pressure conditions, significant component accumulation occurs rapidly within the cylinder. The variation pattern of H_2_O_2_ component is similar to that of CH_2_O. As injection pressure rises, the fuel can more effectively transition from the low-temperature to the medium-temperature zone, leading to a rapid accumulation of H_2_O_2_. Fig 17c displays the variation curve of OH component with fuel injection pressure. The OH concentration significantly increases as injection pressure rises, due to the large accumulation of H₂O₂ during medium-temperature reactions. When the temperature reached 1000 K, the H_2_O_2_ component quickly decomposed into OH component. Subsequently, the OH reacts more rapidly with hydrocarbons, generating even more OH components, resulting in an exponential increase. At 10 deg, the OH content at 140 MPa injection pressure is 68% higher compared to 100 MPa. The abundant presence of OH components can promote faster and more complete oxidation of fuel.

**Fig 18:**
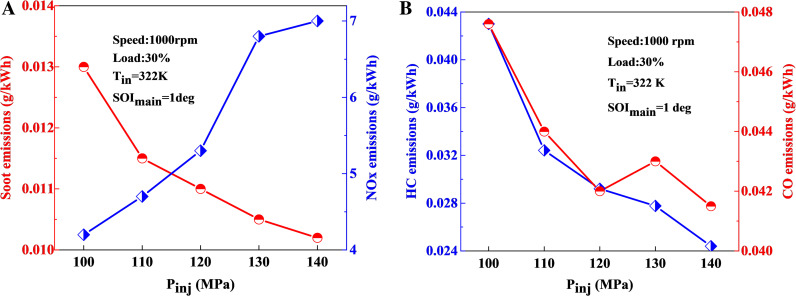
The impact of fuel injection pressure on emissions: (a) soot-NOx; (b) HC-CO.

Fig 18 presents the variation patterns of NOx, CO, HC and soot emissions under different injection pressures at 1000 r/min and 30% load conditions. As the fuel injection pressure increases, the overall emissions of soot, CO and HC show a decreasing trend. This is due to enhanced fuel evaporation, a higher proportion of lean mixtures, an accelerated combustion rate, and increased production of OH components, which facilitate the oxidation and decomposition of incomplete combustion products, thereby reducing emissions of soot, CO, and HC.

However, the abundant presence of OH component also raises the cylinder temperature, resulting in higher NOx emissions. Notably, NOx production increases sharply when the injection pressure exceeds 120 MPa, which poses challenges for NOx emission control. Therefore, a balanced approach is required to optimize the trade-off between reducing incomplete combustion products and controlling NOx emissions, necessitating the selection of an appropriate injection pressure.

The comprehensive analysis of injection pressure’s impact on emissions reveals that both excessively high and low pressures are detrimental to controlling incomplete combustion products. Simply increasing the fuel injection pressure can impose significant mechanical loads on the common rail system. Therefore, considering the advantages and disadvantages, a fuel injection pressure of 120 MPa is selected as optimal.

Under operating condition of 1000 r/min and 30% load, with an ambient temperature o 258 K, an intake temperature of 322 K, and a main injection timing of 1 deg, increasing the injection pressure from 100 MPa to 120 MPa results in a reduction in soot emissions by 21.4%, HC emissions by 30.2%, CO emissions by 11.8%, and an increase in NOx emissions by 23.8%.

Based on the optimization research for conducted under condition of 1000 r/min and 30% load, when the ambient temperature is 258 K, increasing the intake temperature to 322 K, advancing the main injection timing to 1 deg, and raising the injection pressure to 120 MPa, result in a reduction of soot emissions by 23.1%, HC emissions by 33.3%, and CO emissions by 52.3%. However, this optimization also leads to a significant increase in NOx emissions by 116.7% compared to the baseline before optimization.

## 4. Conclusions

A clean combustion mode for heavy-duty diesel engines was proposed based on the spray development process and chemical reaction kinetics. Using an environmental chamber test bench and a numerical simulation platform, the study examined the effects of intake air temperature, injection timing, and injection pressure on combustion and emissions. The spray development, concentration distribution of the fuel-air mixture, and change in combustion intermediates were analyzed to provide technical support for the development of clean combustion system. The main research conclusions are as follows:

1. Increasing intake air temperature, advancing injection timing, and increasing injection pressure enhance the development of in-cylinder spray and fuel-air mixture. Increased fuel evaporation and the reduced SMD of oil droplets promote the transition of liquid fuel to gaseous form, significantly reducing the proportion of locally concentrated areas in the cylinder.
2. Higher intake temperature, advanced injection timing, and increased injection pressure boost the formation of active species critical to combustion, particularly accelerating the generation of H_2_O_2_ components and their conversion to OH radicals. The abundant presence of OH radicals accelerates the oxidation and decomposition of fuel, improving fuel oxidation and reducing incomplete combustion products.
3. Under an ambient temperature of 258 K, a clean combustion strategy involving an intake temperature increase to 322 K, main injection timing advanced to 1 deg, and injection pressure is increased to 120 MPa resulted in significant emissions reductions compared to pre-optimization conditions: soot by 23.1%, HC by 33.3%, and CO by 52.3%.

## Supporting information

S1 File Experimental data at 1000rpm and 30% load conditions. (ZIP)

## Abbreviation

NOx: Nitrogen oxide
HC: Hydrocarbon
CO: Carbon monoxide
Tin: Intake temperature
Pinj: Injection pressure
SOI: Start of injection
SMD: Sauter mean diameter
HRR: Heat release rate; “ - ”, before top dead center
